# Celiac Disease: Cytokine Profile of Intraepithelial Gamma Delta T Cells in Disease Severity

**DOI:** 10.3390/cells15151424

**Published:** 2026-08-06

**Authors:** Giuseppe Mazzarella, Giuseppe Iacomino, Gaetano Iaquinto, Alessandra Camarca, Errico Picariello, Raffaele Melina, Vera Rotondi Aufiero

**Affiliations:** 1 Immuno-Morphology Laboratory, Institute of Food Sciences, National Council Research, 83100 Avellino, Italy; giuseppe.mazzarella@cnr.it (G.M.); giuseppe.iacomino@cnr.it (G.I.);; 2 European Laboratory for the Investigation of Food-Induced Diseases (E.L.F.I.D), University “Federico II”, 80131 Napoli, Italy; 3 Gastroenterology Division, S. Rita Hospital, 83042 Atripalda, Italy; 4 Department of Science and Technology, University of Sannio, 82100 Benevento, Italy; 5 Gastroenterology Department, S. G. Moscati Hospital, 83100 Avellino, Italy

**Keywords:** γδ^+^ intraepithelial lymphocytes, celiac disease, cytokines

## Abstract

**Highlights:**

**What are the main findings?**

- γδ^+^ intraepithelial lymphocytes from active celiac disease display stage-dependent cytokine profiles, with Marsh II samples showing higher IL-10 expression and Marsh III samples showing increased IL-15, IL-17A, IL-21, IFN-γ and TGF-β.
- γδ^+^ IELs are Foxp3^−^ in both Marsh II and Marsh III lesions, indicating that their regulatory-like activity occurs independently of classical Treg differentiation.

**What are the implications of the main findings?**

- γδ^+^ IELs may contribute to intestinal immune regulation in early active disease (Marsh II) through an IL-10–mediated anti-inflammatory response.
- In advanced mucosal damage (Marsh III), γδ^+^ IELs shift toward a pro-inflammatory phenotype, suggesting functional polarization and a potential role in disease progression.

**Abstract:**

γδ^+^ intraepithelial lymphocytes (IELs) are persistently expanded in the intestinal epithelium of patients with active celiac disease (ACeD), but their functional profile during active inflammation remains poorly defined. This study investigated the expression of pro- and anti-inflammatory cytokines in γδ^+^ IELs isolated from the intestinal epithelium of ACeD patients at different stages of mucosal damage. Frozen jejunum sections were obtained from 14 ACeD patients (7 Marsh II and 7 Marsh III) and 10 treated celiac disease (CeD) patients. γδ^+^ IELs from ACeD biopsies and intestinal enterocytes (IEs) from treated CeD biopsies were isolated by laser capture microdissection on mirror sections, followed by RNA extraction and quantitative real-time RT-PCR analysis of IL-15, IL-17A, IL-21, IFN-γ, TNF-α, IL-10, and TGF-β. Foxp3 expression was assessed by immunohistochemistry. γδ^+^ IELs from Marsh III biopsies showed significantly increased mRNA levels of IL-15, IL-17A, IL-21, IFN-γ, and TGF-β compared with IEs, whereas IL-10 expression was significantly higher in Marsh II γδ^+^ IELs compared with Marsh III and IEs. IL-21 and TGF-β were also higher in Marsh III than Marsh II γδ^+^ IELs. All γδ^+^ IELs were Foxp3^−^. These findings indicate a stage-dependent functional polarization of γδ^+^ IELs in ACeD, with an IL-10–associated regulatory profile in Marsh II and a predominant pro-inflammatory cytokine signature in Marsh III.

## 1. Introduction

Celiac disease (CeD) is a chronic, immune-mediated systemic disorder triggered by the ingestion of dietary gluten in genetically predisposed individuals. The immune response against gluten-derived prolamins leads to a complex inflammatory cascade within the small intestinal mucosa, ultimately leading to villous atrophy and crypt hyperplasia. Upon gluten exposure, gliadin-specific CD4^+^ T cells residing in the lamina propria become activated and predominantly polarize toward a T helper 1 (Th1) phenotype, characterized by the production of high levels of pro-inflammatory cytokines such as interferon-γ (IFN-γ) [1]. This Th1-driven immune response represents a central pathogenic mechanism underlying intestinal inflammation and tissue damage in active CeD (ACeD).

The severity of intestinal mucosal injury in celiac disease is commonly classified according to the modified Marsh-Oberhuber classification, which reflects the progressive histopathological changes induced by gluten exposure. Marsh II lesions are characterized by increased intraepithelial lymphocytosis (>25 intraepithelial lymphocytes per 100 enterocytes) associated with crypt hyperplasia while preserving normal villous architecture. In contrast, Marsh III lesions are characterized by intraepithelial lymphocytosis and crypt hyperplasia accompanied by variable degrees of villous atrophy (partial, subtotal, or total), representing the histological hallmark of active celiac disease. These progressive histological changes are associated with increasing mucosal inflammation, epithelial damage, and immune cell infiltration, providing a useful framework for investigating stage-dependent immune responses during disease progression. Since Marsh II and Marsh III represent consecutive stages of active disease with distinct degrees of mucosal damage, they constitute an appropriate model for evaluating whether γδ intraepithelial lymphocytes undergo functional changes during the progression of CeD. This classification is widely adopted for the histopathological diagnosis and staging of celiac disease and has important clinical and research implications [2,3,4].

In addition to the Th1 response, accumulating evidence indicates a significant contribution of Th17 cells to CeD pathogenesis. Increased levels of interleukin-17 (IL-17) have been observed in the inflamed intestinal mucosa of CeD patients, supporting the involvement of Th17-mediated pathways in the pathogenesis of the disease [5,6,7,8]. Gliadin-specific Th17 cells have been shown to produce IL-17 and IL-21 but also transforming growth factor-β (TGF-β), suggesting a dual inflammatory and regulatory potential within this subset [9].

The coexistence of pro-inflammatory and regulatory cytokine production highlights the complexity of immune regulation in CeD and suggests that specific T-cell subsets may exert context-dependent functions during disease progression. Consistent with this concept, increased numbers of Foxp3^+^ Treg cells have been identified in the intestinal mucosa of ACeD patients, possibly reflecting a compensatory mechanism aimed at counteracting ongoing inflammation [10]. In parallel, type 1 regulatory T cells (Tr1), characterized by high production of interleukin-10 (IL-10) and TGF-β, have been implicated in suppressing pathogenic gluten-specific T-cell responses in CeD [10,11].

Notably, Foxp3 expression is not essential for Tr1 cell differentiation or suppressive function, supporting the notion that immune regulation in CeD is not exclusively mediated by classical Foxp3^+^ Treg cells. Indeed, several Foxp3^−^ regulatory T-cell subsets capable of producing IL-10 and TGF-β have also been described [12,13,14].

In this context, γδ T cells have emerged as potent producers of immunoregulatory cytokines, including high levels of TGF-β and IL-10 [15]. These cytokines play a crucial role in maintaining epithelial integrity and intestinal immune homeostasis, and they also contribute to epithelial restitution following tissue injury. Given their strategic localization, γδ T cells are ideally positioned to influence local immune responses at mucosal surfaces.

The intestinal epithelium represents a crucial interface between luminal antigens and the mucosal immune system, and a hallmark of CeD is the marked expansion of IELs, both CD8^+^ TCRαβ^+^ T and TCRγδ^+^ T [16].

While the pathogenic role of CD8^+^ TCRαβ^+^ IELs in mediating epithelial damage has been widely investigated, the biological significance of TCR γδ^+^ IEL expansion, also observed in potential or treated CeD [17,18,19], remains less clearly defined.

Previous studies have reported an increased proportion of γδ^+^ IELs producing TGF-β in patients adhering to a gluten-free diet compared with those with ACeD, pointing to a potential regulatory function of these cells in modulating gluten-reactive Th1 responses [20], which could be restored after the removal of the triggering antigen. However, despite their well-documented expansion in CeD mucosa, the cytokine profile and the immunological role of γδ^+^ IELs across different stages of mucosal damage, as classified according to the Marsh-Oberhuber criteria, remains poorly defined.

To address this gap, we recently developed an optimized laser capture microdissection (LCM) protocol on mirror tissue sections [21], allowing the selective isolation of immunophenotypically identified γδ^+^ IELs while preserving RNA integrity, which is essential for the accuracy and interpretability of downstream transcriptomic analyses. Using this approach, we determined the expression of inflammatory and anti-inflammatory cytokines in γδ^+^ IELs isolated from the intestinal epithelium of patients with ACeD at different Marsh stages by quantitative real-time PCR (qRT-PCR).

The aim of this study is to clarify the functional role of γδ^+^ IELs during the progression of celiac mucosal damage, providing insights into their potential contribution to either amplification or regulation of intestinal inflammation. Understanding whether γδ^+^ T cells exert predominantly inflammatory or regulatory functions at distinct histological stages of mucosal damage may provide critical insights into disease progression and mucosal remodeling.

## 2. Materials and Methods

### 2.1. Patients

Jejunal biopsies were obtained from 14 ACeD patients (3 male and 11 female; median age, 29 years; range, 17–51 years) and immediately snap-frozen in liquid nitrogen. The diagnosis was based on the presence of typical mucosal lesions, including crypt cell hyperplasia and villous atrophy. All ACeD patients were positive for serum antiendomysial antibodies. According to histology severity, patients were classified into two groups: Marsh II ACeD (*n* = 7) and Marsh III ACeD (*n* = 7).

Immunohistochemical data are shown in Table 1.

Jejunal biopsy samples were also obtained from 10 treated CeD patients (1 male and 9 female patients; median age, 48 years; range, 19–56 years). These patients had been following a long-term gluten-free diet, had achieved both clinical and histological remission, and tested negative for anti-endomysial antibodies.

All patients gave written informed consent before enrollment (the informed consent form is available as Supplementary Materials). The study was approved by the ethics committee of San G. Moscati Hospital (Avellino, Italy, n°CECN/809) and was conducted in accordance with the principles of the Declaration of Helsinki.

### 2.2. LCM on Mirror Sections

Fresh-frozen jejunal mucosa samples were mounted on the cryostat stage (Leica CM1850; Leica Microsystems, Wetzlar, Germany) set at −25 °C, allowed to equilibrate for 20 min and processed as previously reported [21]. Briefly, two consecutive mirror cryostat sections (10 μm thickness) were collected on two different glass slides. One section was mounted on a typical glass slide and subsequently processed by immunohistochemical detection of γδ ^+^ cells [22]. The consecutive section was mounted upside down on an RNase-free membrane slide (PEN-membrane, 1 mm glass, Carl Zeiss MicroImaging, Munich, Germany) and processed for LCM [21]. By comparing the IHC-processed sections with the corresponding mirror sections collected on the PEN-membrane slide, approximately 1000 γδ^+^ IELs were microdissected from each ACeD patient, and approximately 1000 IEs were isolated from treated CeD patients as non-inflamed controls, as in active CeD the epithelial layer is extensively infiltrated by lymphocytes, thereby precluding the recovery of an adequate number of enterocytes for downstream molecular analyses.

Due to the very low density of γδ IELs in healthy controls and treated celiac disease, it was not technically feasible to isolate the minimum number of cells required for RNA extraction and downstream RT-qPCR analysis while preserving RNA integrity using LCM. Therefore, the molecular analyses were limited to γδ IELs isolated from ACeD (Marsh II and Marsh III). Enterocytes isolated from treated CeD biopsies (patients on a long-term gluten-free diet with histological remission) were included solely as a non-inflamed epithelial reference. They were not intended as a biological comparator for γδ IELs but were used exclusively to provide baseline transcript expression within the epithelial compartment. Isolation of a pure enterocyte population from active celiac lesions was not feasible because the epithelial layer is extensively infiltrated by intraepithelial lymphocytes, preventing selective epithelial cell capture by LCM.

### 2.3. RNA Extraction, Retrotranscription and RT-qPCR

Microdissected samples were treated with 4 U RNasin RNase inhibitor (Promega, Milan, Italy), and total RNA was obtained using the PicoPure™ RNA isolation kit according to the manufacturer’s instructions (Arcturus, Life Technologies, Carlsbad, CA, USA) were followed. RNA was eluted in 12 μL of DEPC-treated water. RNA concentration was determined using a Qubit^®^ 3 Fluorometer (Thermo Fisher Scientific, Waltham, MA, USA). RNA integrity was assessed by determining the RNA Integrity Number (RIN) using an Agilent 2100 Bioanalyzer system (Agilent Technologies, Santa Clara, CA, USA), according to the manufacturer’s recommendations. Total RNA was reverse transcribed using the SuperScript™ VILO cDNA Synthesis Kit (Life Technologies, Carlsbad, CA, USA) with random hexamer primers, according to the manufacturer’s instructions. Relative cDNA levels of IFN-γ, TNF-α, IL-10, TGF-β, IL-17, IL-21, and IL-15 were quantified by SYBR^®^ Green-based quantitative real-time PCR (Power SYBR^®^ Green PCR Master Mix, Applied Biosystems, Foster City, CA, USA) using an ABI PRISM^®^ 7000 SDS instrument (Applied Biosystems, Foster City, CA, USA). PCR reactions were performed in triplicate in a final volume of 35 μL containing 2 μL of diluted cDNA. Serial dilutions of cDNA containing a known amount of each transcript were included in every quantitative PCR run to generate standard curves. Amplification specificity was verified by melting-curve analysis. Only samples in which both target and housekeeping genes displayed a sigmoidal amplification curve with Ct values between 15 and 36 were included in the analysis. Samples failing to meet RNA quality or quantity requirements were excluded from the study.

Primer pairs were designed using Primer Express Software (Version 3.0.1, Applied Biosystems, Foster City, CA, USA) in accordance with ABI guidelines or were derived from published literature and were synthesized by Sigma-Aldrich (St. Louis, MI, USA). Primers were designed to generate amplicons of approximately 50–150 bps from specific National Center for Biotechnology Information (NCBI) reference sequences, to span exons, and were blasted through NCBI GenBank to exclude homology with unrelated human cDNA sequences.

Cycling conditions for RT-qPCR have been described elsewhere [23]. Gene expression levels were normalized to the level of the housekeeping gene glyceraldehyde-3-phosphate dehydrogenase (GAPDH). The primer sequences are reported in Table 2.

### 2.4. Immunohistochemistry

Acetone-fixed sections (5 μm) from biopsy samples were used to perform staining experiments to characterize γδ T lymphocytes using specific antibodies against TCR γδ in combination with Foxp3. Costaining experiments were set up by mixing rat anti-human Foxp3 with mouse anti- TCR γδ and incubating the cryosections for 1 h. After incubation with primary antibodies, the cryosections were incubated with a mixture of goat anti-rat tetramethyl-rhodamine isothiocyanate-conjugated antibody and goat anti-mouse fluorescein isothiocyanate-conjugated antibody for 30 min. Finally, all the sections were counterstained with DAPI (Molecular Probes, Leiden, The Netherlands), mounted in phosphate-buffered saline:glycerol (1:1), and imaged with a Leica SP8 confocal microscope (Leica Microsystems, Heidelberg, Germany). Quantitative analyses were conducted in multiple regions of the epithelium and lamina propria. The spatial distribution and co-expression of markers were assessed across the different Marsh grades. Non-immune mouse immunoglobulins were used as isotype controls for primary antibodies. The density of cells expressing Foxp3 in the lamina propria was evaluated within a total area of 1 mm^2^ of lamina propria. Slides were independently analyzed by two blind observers.

### 2.5. Data and Statistical Analysis

Relative transcript expression was calculated by the ΔΔCT method with Data Assist Software v3.01 (Applied Biosystems). Statistical analyses were performed using GraphPad Prism 6 Software (GraphPad Software, San Diego, CA, USA). Differences between groups were assessed using unpaired two-tailed Student’s *t*-tests. Statistical significance was defined as *p* < 0.05. Results are reported as normalized mean expression levels.

## 3. Results

### 3.1. Predominant Pro-Inflammatory Immune Response of γδ^+^ IELs in Marsh III Lesions

To evaluate the expression profile of inflammatory cytokines, we first analyzed the mRNA expression of IL-15, IL-17A, IL-21, IFN-γ, and TNF-α in γδ^+^ IELs isolated by LCM from jejunal biopsies of Marsh II and Marsh III patients. These data were compared with non-inflamed epithelial reference obtained from treated CeD patients.

**Figure 1:**
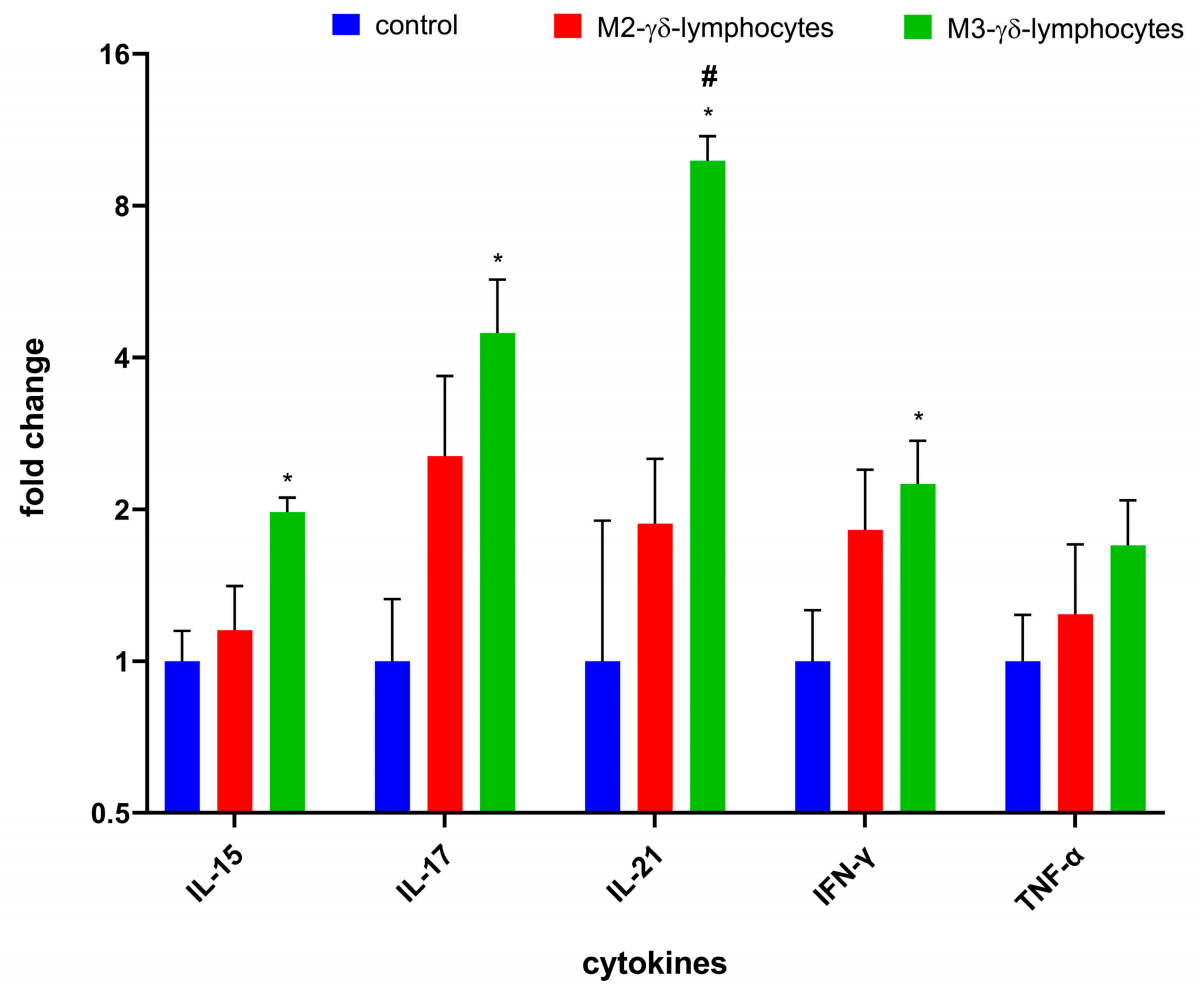
Inflammatory cytokine expression by γδ^+^ IELs from ACeD patients. Selected mRNA transcripts were analysed by RT-qPCR in γδ^+^ intraepithelial lymphocytes (IELs) isolated by immuno-LCM from jejunal biopsies of ACeD patients with Marsh II (M2) (red bar) and Marsh III (M3) (green bar) lesions (*n* = 7 per group). Fold change (y-axis) represents mRNA expression normalized to the GAPDH reference gene. Transcript levels were expressed relative to those in non-inflamed epithelial tissue (blue bar). Statistically significant differences are indicated by the following symbols: * *p* < 0.05 versus the control group; # *p* < 0.005 versus the M2 group.

When γδ^+^ IELs isolated from Marsh III biopsy specimens were compared with IEs isolated from treated CeD controls, all analysed pro-inflammatory cytokines were significantly upregulated, except for TNF-α, which showed an increasing trend without reaching statistical significance (Figure 1). A non-significant increasing trend was also observed for all the investigated cytokines when comparing γδ^+^ IELs isolated from Marsh II biopsies with IEs isolated from treated CeD patients (Figure 1). These findings support the concept that the fully established pro-inflammatory cytokine milieu is primarily associated with advanced mucosal damage.

Within the ACeD cohort, γδ^+^ IELs isolated from Marsh III biopsy specimens showed higher mRNA levels of IL-15, IL-17A, IFN-γ, and TNF-α compared with γδ^+^ IELs isolated from Marsh II biopsies, although these differences did not reach statistical significance (Figure 1). Notably, we observed that only IL-21 expression was significantly higher in γδ^+^ IELs isolated from Marsh III lesions compared to γδ^+^ IELs isolated from Marsh II biopsy specimens (*p* < 0.05) (Figure 1), suggesting that γδ^+^ IELs associated with severe mucosal lesions preferentially acquire a Th17-like pro-inflammatory profile.

### 3.2. Predominant Regulatory Immune Response of γδ^+^ IELs in Marsh II Lesions

To further define the regulatory versus inflammatory balance of γδ^+^ IELs, we analyzed the expression of the anti-inflammatory cytokines IL-10 and TGF-β.

**Figure 2:**
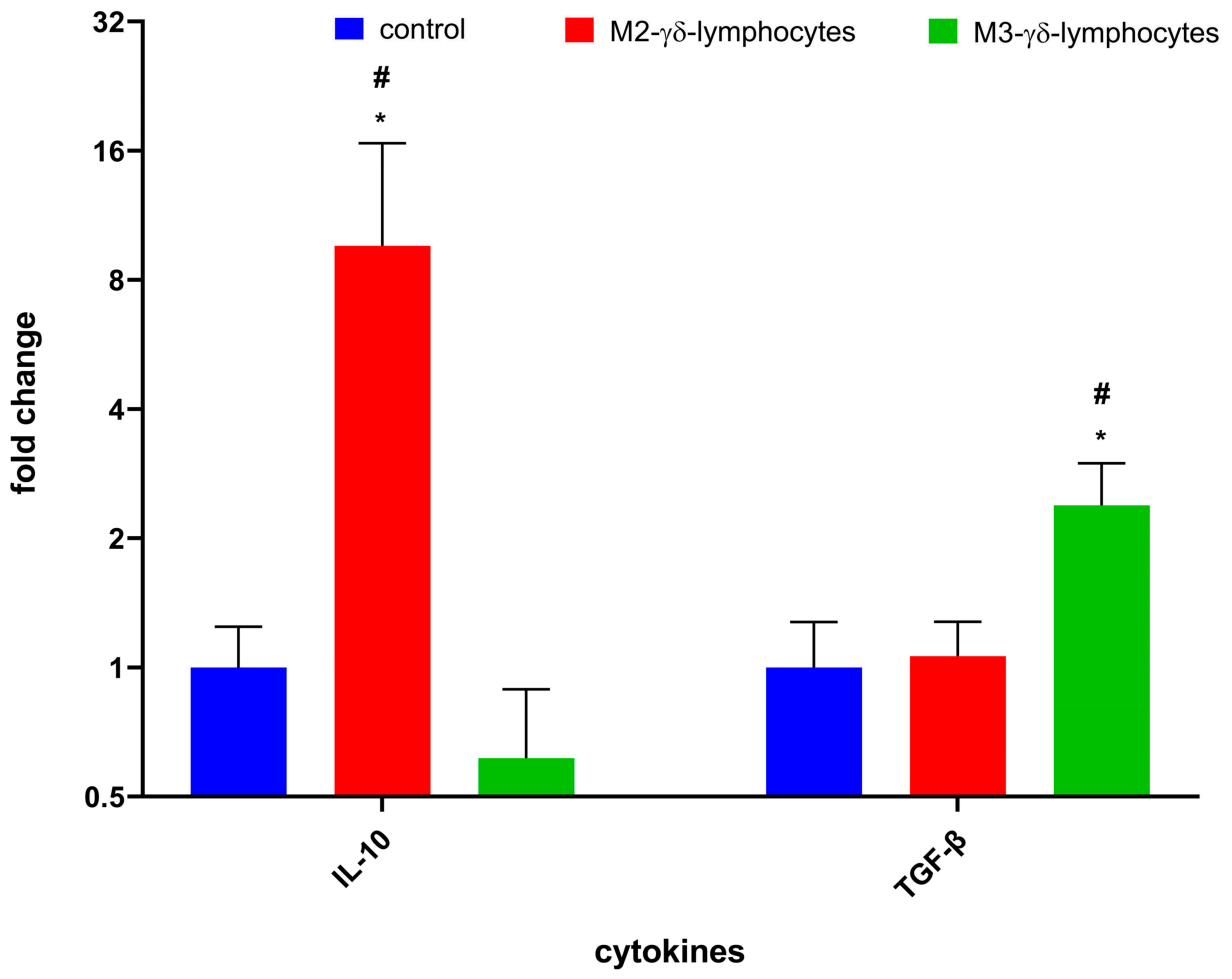
Anti-inflammatory cytokine expression in γδ^+^ IELs from ACeD patients. Selected mRNA transcripts were analysed by RT-qPCR in γδ^+^ intraepithelial lymphocytes (IELs) isolated by immuno-LCM from jejunal biopsies of ACeD patients with Marsh II (M2) (red bar) and Marsh III (M3) (green bar) lesions *(n* = 7 per group). Fold change (y-axis) represents messenger RNA expression normalized to the GAPDH reference gene. mRNA expression was normalized to that of non-inflamed epithelial tissue (blue bar). Statistically significant differences are indicated by the following symbols: * *p* < 0.05 versus the control group; # *p* < 0.005 M3 versus the M2 group.

Both IL-10 and TGF-β transcripts were detected in γδ^+^ IELs isolated from Marsh II and Marsh III biopsy specimens. However, IL-10 mRNA levels were significantly higher in γδ^+^ IELs isolated from Marsh II mucosae compared with those isolated from Marsh III biopsy specimens and with IEs isolated from treated CeD (*p* < 0.005 and *p* < 0.05, respectively) (Figure 2). These findings suggest that in Marsh II lesions, γδ^+^ IELs may exert a more pronounced immunoregulatory function, potentially contributing to immune modulation through a Treg-associated cytokine profile.

In contrast, TGF-β expression was significantly increased in Marsh III γδ^+^ IELs compared to both γδ^+^ IELs isolated from Marsh II and IEs specimens isolated from treated CeD (*p* < 0.005 and *p* < 0.05, respectively) (Figure 2).

### 3.3. Immunoregulation Activity Involves γδ IELs/Foxp3^−^ in ACeD Patients

To further characterize the immunoregulatory activity of γδ IELs, we performed immunohistochemical analyses using antibodies specific for Foxp3, a transcription factor that plays a central role in the development and function of regulatory T cells (Tregs).

As previously reported [7], Foxp3^+^ exhibit a characteristic nuclear localization in positive cells and were detected exclusively within the lamina propria, predominantly in the subepithelial compartment, with no detectable expression in the epithelial layer.

**Figure 3:**
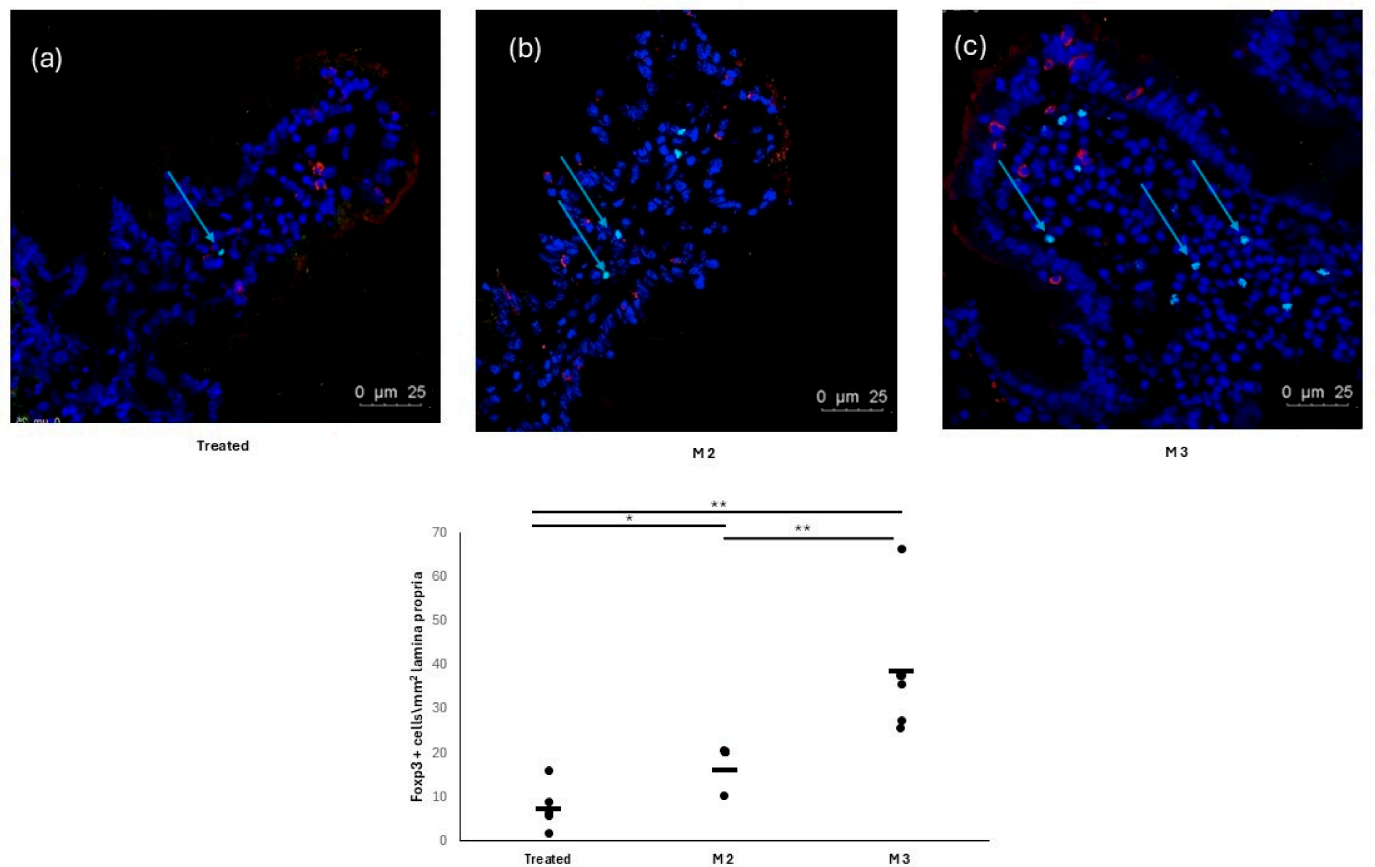
Increased Foxp3^+^ cells in the lamina propria of Marsh III CeD and lack of Foxp3 expression in γδ intraepithelial lymphocytes. (**Upper panel**): Phenotyping of forkhead box P3 (Foxp3^+^) cells in jejunal biopsy samples from treated CeD (**a**), Marsh II (M2) (**b**), and Marsh III (M3) (**c**) patients. Cyan arrows indicate representative Foxp3^+^ cells. Immunofluorescence co-staining experiments revealed that all γδ^+^ cells were Foxp3-negative. Red indicates γδ molecule staining, cyan indicates Foxp3 molecule staining, and blue indicates nuclear counterstaining with To-Pro-3. Original magnification: ×40. (**Lower panel**): Number of forkhead box P3 (Foxp3^+^) cells analysed by immunohistochemistry in mucosal explants from treated CeD, M2, and M3 patients. Foxp3^+^ cells were counted per square millimetre of the lamina propria. Dots represent single patients. Dashes indicate the mean values. Statistical significance was assessed by comparing M2 and M3 with treated CeD, and M2 with M3 patients. * *p* < 0.05; ** *p* < 0.005.

Immunofluorescence co-staining demonstrated the localization of Foxp3^+^ cells within the lamina propria in treated CeD, Marsh II and Marsh III biopsies (Figure 3a–c, upper panel, cyan arrows).

Representative merged confocal images also confirmed that γδ^+^ intraepithelial lymphocytes (red spots) did not co-express Foxp3. Accordingly, γδ^+^ IELs localized within the intestinal epithelium were consistently Foxp3-negative in all Marsh II and Marsh III specimens analyzed (Figure 3a–c, upper panel).

Quantitative analysis showed a significantly higher density of Foxp3^+^ cells in the lamina propria of Marsh III patients compared with treated CeD and Marsh II patients (Figure 3, lower panel).

Patients with Marsh III lesions showed a significantly higher density of Foxp3^+^ cells in the lamina propria (*p* < 0,05; Figure 3c, upper panel) compared with those with Marsh II lesions (*p* < 0,05; Figure 3b, upper panel). Overall, the increased density of Foxp3^+^ cells appear to correlate with the severity of the histological lesion. The increased area of the lamina propria in Marsh III lesions reflects the inflammatory expansion associated with severe villous atrophy. To avoid potential sampling bias, Foxp3-positive cells were expressed as cell density (cells/mm^2^ of lamina propria) instead of absolute cell numbers.

We found that in Marsh II and Marsh III CeD patients, the number of Foxp3-expressing cells per mm^2^ in the lamina propria was significantly higher (mean ± s.d.: 15.36 ± 7.24 vs. 37.73 ± 10.28 cells/mm^2^, respectively) compared with treated CeD (mean ± s.d.: 7.2 ± 4.6; *p*< 0.005) (Figure 3, lower panel).

## 4. Discussion

In CeD, γδ T cells possess the ability to produce anti-inflammatory cytokines, therefore contributing to epithelial homeostasis and tissue repair [20]. However, whether this regulatory paradigm is maintained across different histological stages of active disease has not been systematically investigated.

In this study, we addressed this issue for the first time by conducting a compartment-specific cytokine analysis of γδ^+^ IELs across different histological stages of ACeD (Marsh II and Marsh III) using a combined LCM and RT-qPCR approach. The use of a mirror-section LCM protocol [21] allowed the precise identification and selective isolation of γδ^+^ IELs from immunostained sections while preserving RNA integrity in the corresponding unstained mirror sections.

The mirror-section strategy was specifically implemented to minimize cross-contamination from adjacent epithelial cells. Although a certain degree of contamination cannot be completely excluded in any microdissection-based approach, the selective identification and isolation of individual γδ-positive IELs considerably reduces this risk compared with conventional whole-biopsy analyses.

One limitation of this approach is that the analysis could be restricted to Marsh II and Marsh III lesions, as in Marsh 0 and Marsh I samples, the density of γδ^+^ IELs was too low to allow the isolation of an adequate number of cells. Specifically, at least 1000 cells needed to be collected within a maximum time window of 30 min, a prerequisite to preserve RNA integrity and ensure reliable RT-qPCR quantification. A limitation of the present study is the lack of molecular analyses of γδ intraepithelial lymphocytes from early histological stages, healthy controls or treated celiac disease. Although the inclusion of these comparator groups would have strengthened the study, the low density of γδ IELs in non-active mucosa made it technically unfeasible to isolate a sufficient number of cells by laser capture microdissection while preserving RNA integrity for downstream gene expression analyses. Consequently, the present study was specifically designed to investigate whether γδ IELs undergo functional changes during the progression of ACeD by comparing Marsh II and Marsh III lesions, rather than to identify differences between active and non-active disease. Future studies using single-cell transcriptomics or other high-sensitivity approaches may help overcome this limitation and further characterize γδ IELs across the entire spectrum of CeD.

Given the central role of epithelial danger signals in driving IEL activation and functional polarization, we investigated whether IL-15, a key driver of IEL licensing in CeD, was differentially expressed in γδ^+^ IELs across disease stages. In line with previous studies identifying IL-15 as a master regulator of IEL activation and cytotoxic programming [24,25], we observed increased IL-15 expression in γδ^+^ IELs, particularly in Marsh III lesions. IL-15 is known to lower activation thresholds, enhance cell survival, and potentiate effector functions in tissue-resident lymphocyte populations, thereby supporting a role for IL-15–driven signaling in shaping the pro-inflammatory phenotype of γδ^+^ IELs at advanced stages of mucosal damage. In CeD, IL-15 has been classically described as being predominantly produced by epithelial cells and antigen-presenting cells within the mucosa, where it acts as a key epithelial “danger” signal. Consistent with this paradigm, compartment-based analyses have shown that IL-15 is selectively upregulated within the epithelial compartment of the small intestinal mucosa of CeD patients [23]. Notably, our compartment-specific approach revealed that γδ^+^ IELs themselves express IL-15 transcripts, suggesting that these cells may represent, beyond enterocytes, an additional intraepithelial source of IL-15, particularly during advanced mucosal damage.

This observation raises the possibility that γδ^+^ IELs actively contribute to sustaining IL-15 availability within the epithelial niche, potentially reinforcing pro-inflammatory functional polarization and promoting the persistence of activated IELs.

In this context, the increased expression of TGF-β observed in γδ^+^ IELs in Marsh III specimens may not necessarily indicate the engagement of a classical regulatory program but could instead reflect a component of a complex and dysregulated inflammatory microenvironment. The potential dual or context-dependent role of TGF-β in advanced disease stages will be discussed further below.

In addition to IL-15, we analyzed the expression of other pro-inflammatory cytokines and found that γδ^+^ IELs isolated from both Marsh III and Marsh II subjects showed higher mRNA levels of IL-21, IL-17A, IFN-γ and TNF-α relative to the non-inflamed epithelial reference obtained from treated CeD enterocytes. However, a statistically significant upregulation of these cytokines was observed only in γδ^+^ IELs from Marsh III compared with the epithelial reference. It should be emphasized that enterocytes isolated from treated celiac disease were included solely as a non-inflamed epithelial reference and not as a biological comparator for γδ^+^ IELs. Therefore, these comparisons should be interpreted as differences between distinct cellular compartments rather than disease-specific changes within the same cell population. A particularly relevant observation was the significant upregulation of IL-17 and IL-21 in Marsh III γδ^+^ IELs. Several studies have highlighted the involvement of Th17-type immune response in CeD pathogenesis, as evidenced by the increased expression of Th17-associated cytokines in patients with ACeD [26]. Furthermore, gliadin-specific Th17 cells have been shown to produce not only IL-17, but also IL-21 and IFN-γ, together with the mucosa-protective cytokine IL-22 and the regulatory cytokine TGF-β. These observations indicate that Th17 cells in CeD are functionally plastic, capable of secreting both pro-inflammatory and regulatory mediators depending on the immune microenvironment. Although gluten-specific HLA-DQ2–restricted CD4^+^ T cells are considered a major source of IL-17 and IL-21 in ACeD [27], our data suggest that γδ^+^ IELs may also contribute to the local IL-17 and IL-21 pool within the epithelial compartment at advanced stages. This additional intraepithelial source of IL-17 and IL-21 may further amplify the inflammatory microenvironment and contribute to the persistence of mucosal inflammation.

Our findings should also be interpreted in light of recent transcriptomic studies investigating γδ T cells in CeD using bulk and single-cell RNA sequencing approaches [28,29]. These high-throughput technologies provide an unbiased and comprehensive characterization of γδ T-cell transcriptional programs and cellular heterogeneity at a much broader resolution than the targeted RT-qPCR approach adopted in the present study. Nevertheless, our LCM-based strategy offers the unique advantage of selectively analyzing γδ IELs within their native tissue compartment, minimizing contamination from neighboring epithelial and lamina propria cells. Therefore, differences between our findings and those reported by transcriptomic studies may reflect not only the different analytical platforms employed but also differences in tissue compartment, disease stage, and cellular populations analyzed.

The kinetics of regulatory cytokines further underscore the stage-specific nature of γδ^+^ IEL function. While the majority of human intestinal Tregs are CD4^+^Foxp3^+^ and are predominantly localized in the lamina propria, distinct subsets of γδ^+^ IELs have also been proposed to contribute to mucosal immune regulation at the epithelial interface.

In CeD, small intestinal γδ^+^ IELs have been reported to display regulatory potential, with enhanced secretion of TGF-β following ligation of inhibitory receptors such as NKG2A. This mechanism has been shown to modulate the cytotoxic activity of neighboring αβ IELs, thereby limiting epithelial damage and contributing to mucosal immune homeostasis [20].

These observations support the concept that γδ^+^ IELs are capable of stage-dependent immune regulation, complementing classical CD4^+^ Treg-mediated mechanisms and contributing to epithelial homeostasis.

Consistent with these findings, we observed that γδ^+^ IELs from both Marsh II and Marsh III lesions express IL-10 and TGF-β, suggesting that a subset of these cells may actively contribute to a Treg-like response, aimed at limiting inflammation. Notably, this regulatory activity appears to be mediated by γδ^+^ Foxp3^−^ cells. IL-10 expression was significantly higher in Marsh II compared to Marsh III lesions, suggesting that γδ^+^ IELs may initially engage in a compensatory anti-inflammatory response during intermediate stages of mucosal injury. By contrast, despite increased TGF-β expression in Marsh III lesions, IL-10 levels were markedly reduced, suggesting that these regulatory mediators follow distinct, context-dependent trajectories during disease progression.

In this regard, it has been shown that TGF-β can promote regulatory T-cell differentiation in isolation, whereas, in the presence of pro-inflammatory cytokines, including IL-21, it may contribute to Th17 polarization [30,31]. Thus, the increased TGF-β that we observed in Marsh III γδ^+^ IELs, along with the increase in IL-17 and IL-21, may not necessarily reflect a regulatory program but could instead represent a component of a broader pro-inflammatory milieu, perhaps promoting a Th17-type response.

Taken together, our findings demonstrate that γδ^+^ IELs exhibit a dynamic, stage-dependent cytokine profile, retaining the capacity to express both pro- and anti-inflammatory mediators. This is the first study to perform a compartment-specific transcriptional analysis of laser-captured γδ IELs according to the histological stage of active celiac disease, highlighting their stage-dependent cytokine profile and suggesting a functional remodeling during disease progression.

## 5. Limitations

This study has some limitations that should be acknowledged. First, the analysis was performed on intestinal tissue obtained from patients with celiac disease, and the inclusion of intestinal biopsies from otherwise healthy individuals as a control group is ethically challenging and generally not feasible, as obtaining invasive tissue samples solely for research purposes is not ethically justified in healthy subjects. Therefore, the lack of intestinal tissue from healthy controls represents an inherent limitation of studies investigating human intestinal mucosal immune responses. To partially address this limitation, our findings were interpreted in the context of the established literature on intestinal γδ^+^ intraepithelial lymphocytes and celiac disease. In addition, the relatively limited sample size, particularly when considering the stratification of patients according to disease severity, may have reduced the statistical power to detect differences in some cytokine transcripts. Larger, multicenter studies and the development of advanced human intestinal models, including patient-derived intestinal organoids and organoid-based co-culture systems, may help overcome some of these limitations and further clarify the contribution of γδ^+^ IELs to the pathogenesis and progression of celiac disease.

## 6. Conclusions

Our findings suggest that γδ IELs undergo stage-dependent functional remodeling during active CeD and may contribute to both inflammatory and regulatory pathways according to the severity of mucosal damage, with advanced Marsh III lesions being characterized by increased expression of IL17A, IL21, IFNγ, TNFα, and IL15 transcripts, implicating these cytokines in the pathogenesis of epithelial inflammation.

At the same time, γδ^+^ IELs retain the capacity to express immunoregulatory mediators. Specifically, IL10 expression is more prominent at earlier disease stages (Marsh II), while TGFβ remains detectable also in Marsh III lesions. This pattern supports the concept of stage-dependent functional remodeling of γδ^+^ IELs rather than a unidirectional and uniform shift toward inflammation.

Further studies are warranted to clarify whether functionally distinct γδ^+^ IEL subsets coexist with either pro-inflammatory or regulatory functions, or whether individual γδ^+^ IELs exhibit functional plasticity, enabling the simultaneous production of cytokines with opposing biological effects.

A deeper understanding of γδ^+^ T-cell biology in intestinal homeostasis and CeD pathogenesis may provide novel insights into disease mechanisms and support the identification of new targets for therapeutic intervention.

## Figures and Tables

**Table 1:** Immunohistochemical quantification of γδ^+^ intraepithelial lymphocytes according to Marsh grade.

Patient	Marsh Grade	γδ mm ep
#1	III	20.8
#2	III	24.6
#3	III	26.8
#4	III	46.5
#5	III	32.7
#6	III	40.2
#7	III	43.6
#8	II	31.5
#9	II	43
#10	II	24
#11	II	25
#12	II	30
#13	II	25
#14	II	27

**Table 2:** PCR primers used in cytokine profiling of celiac mucosa-.

Gene	Accession Number	FWR Primer (Sequence 5’ → 3’)	REV Primer (Sequence 5’ → 3’)
IL-10	AY029171.1	GCTGGAGGACTTTAAGGGTTACCT	CTTGATGTCTGGGTCTTGGTTCT
IL-15	U14407	CCATCCAGTGCTACTTGTGTTTACTT	CCAGTTGGCTTCTGTTTTAGGAA
IL-17A	NM_002190	CAATCCCACGAAATCCAGGATG	GGTGGAGATTCCAAGGTGAGG
IL-21	NM_021803	CATGGAGAGGATTGTCATCTGTC	CAGAAATTCAGGGACCAAGTCAT
IFN-γ	NM_000619	GTTTTGGGTTCTCTTGGCTGTTA	AAAAGAGTTCCATTATCCGCTACATC
TNF-α	NM_000594	CCCCAGGGACCTCTCTCTAATC	GGTTTGCTACAACATGGGCTACA
TGF-β1	NM_000660	CAAGGGCTACCATGCCAACT	AGGGCCAGGACCTTGCTG
GAPDH	NM_002046.2	ATGACATCAAGAAGGTGGTG	CATACCAGGAAATGAGCTTG

## Data Availability

The data presented in this study are available from the corresponding author upon request.

## Supplementary Materials

The following supporting information can be downloaded at: https://www.mdpi.com/article/10.3390/cells15151424/s1.

## Abbreviations

The following abbreviations are used in this manuscript:

IELs	Intraepithelial lymphocytes
ACeD	Active celiac disease
CeD	Celiac disease
IEs	Intestinal enterocytes
Th1	T helper 1
IFN-γ	Interferon-γ
IL-17	Interleukin-17
TGF-β	Transforming growth factor-β
Tr1	Type 1 regulatory T cells
IL-10	Interleukin-10
LCM	Laser capture microdissection
qRT-PCR	Quantitative real-time PCR
GAPDH	Glyceraldehyde-3-phosphate dehydrogenase
Foxp3	Forkhead box P3
