# Supplementary material for: Celiac Disease: Cytokine Profile of Intraepithelial Gamma Delta T Cells in Disease Severity
Source: Cells. 2026 Aug 6;15(15):1424. doi: 10.3390/cells15151424 (PMC13465715; doi:10.3390/cells15151424)
Supplement: Supplementary file 1 [file cells-15-01424-s001.zip › cells-4343043-supplementary.pdf]

SAN GIUSEPPE MOSCATI - AVELLINO
AZIENDA OSPEDALIERA DI RILIEVO NAZIONALE E DI ALTA SPECIALITÀ

EXPRESSION AND ACQUISITION OF INFORMED CONSENT FOR THE COLLECTION AND USE OF INTESTINAL BIOPSIES FOR SCIENTIFIC RESEARCH PURPOSES

Declarant

I, the undersigned ______________________________, born in ______________________________

on ______________, residing in ___________________________________
Street _______________________________________

having been informed by Prof./Dr. ____

- about the use of my intestinal biological samples, already collected during routine medical practice, exclusively for scientific purposes (for which confidentiality of personal data is guaranteed and may be used for possible scientific publications). In particular, these biopsies will be used for the research project:

“Immunomodulatory function of γδ+ intraepithelial lymphocytes in Celiac Disease: in vivo and in vitro responses to gliadin stimuli”,
winner of the **GRANT FC FELLOWSHIP 2017**.

Having evaluated the information received and the clarifications provided to me, and having understood what is briefly stated above:

I agree to the performance of intestinal biopsy sampling and to their subsequent use for scientific research purposes.

Date ____________________ Signature _________________________________

Signature of the Gastroenterologist Physician
____

All collected information, and in particular personal data, is protected by Legislative Decree No. 196/2003 concerning the “Protection of individuals and other subjects with regard to the processing of personal data”.

We thank you for your attention in reading these notes and kindly ask you to date and sign this form to acknowledge receipt and understanding.

Date ____________________ Signature _________________________________
